# Wearable Technology to Quantify the Nutritional Intake of Adults: Validation Study

**DOI:** 10.2196/16405

**Published:** 2020-07-22

**Authors:** Sarah M Dimitratos, J Bruce German, Sara E Schaefer

**Affiliations:** 1 Foods for Health Institute University of California Davis, CA United States

**Keywords:** wearable technology, mobile health, mobile phone, food intake, validation study

## Abstract

**Background:**

Wearable and mobile sensor technologies can be useful tools in precision nutrition research and practice, but few are reliable for obtaining accurate and precise measurements of diet and nutrition.

**Objective:**

This study aimed to assess the ability of wearable technology to monitor the nutritional intake of adult participants. This paper describes the development of a reference method to validate the wristband’s estimation of daily nutritional intake of 25 free-living study participants and to evaluate the accuracy (kcal/day) and practical utility of the technology.

**Methods:**

Participants were asked to use a nutrition tracking wristband and an accompanying mobile app consistently for two 14-day test periods. A reference method was developed to validate the estimation of daily nutritional intake of participants by the wristband. The research team collaborated with a university dining facility to prepare and serve calibrated study meals and record the energy and macronutrient intake of each participant. A continuous glucose monitoring system was used to measure adherence with dietary reporting protocols, but these findings are not reported. Bland-Altman tests were used to compare the reference and test method outputs (kcal/day).

**Results:**

A total of 304 input cases were collected of daily dietary intake of participants (kcal/day) measured by both reference and test methods. The Bland-Altman analysis had a mean bias of −105 kcal/day (SD 660), with 95% limits of agreement between −1400 and 1189. The regression equation of the plot was Y=−0.3401X+1963, which was significant (*P*<.001), indicating a tendency for the wristband to overestimate for lower calorie intake and underestimate for higher intake. Researchers observed transient signal loss from the sensor technology of the wristband to be a major source of error in computing dietary intake among participants.

**Conclusions:**

This study documents high variability in the accuracy and utility of a wristband sensor to track nutritional intake, highlighting the need for reliable, effective measurement tools to facilitate accurate, precision-based technologies for personal dietary guidance and intervention.

## Introduction

Diet and health guidelines are based on preventing or treating illnesses in the general population. Technological advances and enhanced understanding of systems biology are guiding scientists to pursue personalized interventions for disease prevention and treatment. As scientists are quantifying the elasticity of human health and its diversity, opportunities to intervene in human health are broadening to include precision control of phenotypic performance. Precision or personalized health is the approach of using quantified information on individual characteristics to develop tailored products and services aimed at guiding the underlying processes of health [1-5]. The breadth of precision interventions includes the measurement of individuals’ characteristics, genetics, immunity, metabolism, physiology, medical history, and more [6-8]. Personalizing the content and delivery of approaches also require alignment with individuals’ behaviors, preferences, goals, and barriers to modification as an integral aspect of achieving lasting behavior change [2,9,10].

Precision health is made possible by modern tools, technologies, and platforms that provide increasingly diverse, mechanistic, and accurate assessments of the human body [11,12]. Health measurement research encompasses the breadth of phenotypic differences between individuals that contribute to health status. Advancements in the -omics sciences highlight how many factors individually and interactively affect health, including genetics, lifestyle, life stage, diet, and microbial diversity. Many health metrics are assessed statically, but others must be captured dynamically using specific challenges, such as with insulin sensitivity and acquired immunity. These scientific breakthroughs are guiding the development of measurement technologies that interrogate individuals beyond disease diagnostics, including mobile and wearable body sensors that enable more spatially and temporally specific measures of a broader range of phenotypic factors [4,13-15].

The most important change in the science of diet and health is as much philosophical as mechanistic. The focus of nutrition research is shifting from the study of individual foods and ingredients and their effects on entire populations to the study of individual humans in response to entire diets.

### Precision Nutrition: Challenges and Breakthroughs

Bringing accurate health monitoring technologies to the market provides a public service that reduces people’s uncertainty about how day-to-day choices affect their individual health [15,16]. More precise and predictive dietary guidance follows the understanding in nutritional sciences that identifying the single *best diet* for human health is no longer scientifically defensible. It is now understood that different people respond differently to foods and nutrients, warranting personalized approaches to nutrition interventions and services [3,17-21]. National dietary guidelines are intended to prevent deficiency and maintain health for the majority of the population. Using evidence-based science to create diets for individuals requires an understanding of what humans share with regard to dietary needs as well as how, when, and why needs differ. On a fundamental level, all people require a diet sufficient in calories to support normal body weight and all essential nutrients to support life. However, nutrient requirements to prevent deficiency and sustain life are just the first step in understanding the role of diet in human health [22].

A fundamental challenge in nutrition research is the accurate quantification of food intake and its interpretation as precise diet quality. Currently, the gold standard of dietary assessment is the 24-hour in-patient study, yet major limitations include cost, reduced physical activity, boredom, depression, and weight loss because of reduced dietary freedom and food options deviating from one’s personal routine. In epidemiologic and clinical nutrition, dietary assessment typically relies on researcher-facilitated or autonomous participant recall using methods such as 24-hour recall, food frequency questionnaires, and food diary inventories. These memory-based assessment methods have demonstrated poor validity because of human under- or overestimation of intake and intentional or unintentional alteration of intake patterns [23]. Each traditional assessment method is a reflection of the individual’s perceived intake rather than an accurate measure of true intake. Furthermore, such assessment methods are nonfalsifiable, as what the participant reports must be accepted as truth, despite knowledge of likely incongruence. Moreover, other assessment methods rely on photograph analysis of foods consumed, conducted either by trained personnel or software analysis [24]. Although more closely reflective of nutrient intake in a free-living situation, the remote food photography method is still limited by the inability to record in *true* real time, difficulty in estimating portion sizes, the necessity for simultaneous use of a backup analysis method, difficulty analyzing culturally unique foods, and analyzing mixed dishes via photographs alone. The United States Department of Agriculture (USDA) Food Composition Database is the gold standard for nutrient analysis; albeit comprehensive in scope, this tool cannot possibly account for inherent variability in climate, soil quality, geographic location, item ripeness, and cooking method, all of which may significantly alter the nutrient composition of food. Even nutrient and energy quantification by way of nutrition facts label analysis is error prone, as the Food and Drug Administration allows for certain margins of error in nutrient reporting on packaged food labels. Nutrition fact labels, therefore, provide an educated estimation of packaged food nutrient content. Consumer-focused dietary tracking methods use databases that are often crowdsourced. These errors, compounded with the aforementioned human misreporting of dietary intake, demonstrate that more precise methods of dietary assessment and analysis are needed.

Standard approaches for recording dietary intake do not account for inherent nutrient losses in absorption and metabolism, the transformative processes by which food becomes usable energy for the body. Realistic and precise quantitative assessment remains challenging because of energy losses involved at every step of transforming a food matrix into bioavailable energy: absorption, distribution, metabolism, and excretion. The rate of breakdown and net usable energy vary depending on macronutrient composition (ie, a mixed meal high in fiber, protein, and fat will digest much more slowly than a meal high in simple carbohydrates) [25]. Furthermore, interindividual differences in metabolic rate, gastrointestinal health, and previous meals consumed all contribute to discrepancies between measured intake and bioavailable energy.

Emerging commercial and medical technologies designed to detect a person’s physiological fluctuations claim to capture more dynamic aspects of cardiometabolic health [14]. For example, continuous glucose monitors are designed to provide more precise tracking of glucose levels for diabetic patients compared with standard blood sampling methods, the goal being to more precisely guide disease management [26,27]. No technologies are available that can effectively assess dietary intake directly, although some methods are claiming the ability to estimate dietary intake by assessing the physiological response of the body to food intake and bioavailable energy. In all cases, rigorous testing is necessary to determine the accuracy, precision, utility, and validity of candidate devices. We sought to answer the question, “can wearable technologies measure aspects of metabolic performance and cardiometabolic health of a normal range of adult human phenotypes?” The objectives of this paper were to describe (1) the development and implementation of a reference method to estimate the nutritional intake of free-living study participants and (2) the accuracy and utility of a wristband technology for tracking nutritional intake (kcal/day).

## Methods

### Overview

A study was designed to assess the ability of wearable technology to estimate the nutritional intake of individuals. The wristband (GoBe2; Healbe Corp) intends to provide users with automatic tracking of daily energy intake (calories) and macronutrient intake (grams of protein, fat, and carbohydrates). The technology uses computational algorithms to convert bioimpedance signals into measured patterns of extracellular and intracellular fluids associated with the influx of glucose and essential nutrients into the body. From changes in fluid concentration, the technology estimates calories congruent with glucose absorption into the bloodstream. Time series data such as these, which capture postprandial processes, have the potential to inform phenotypic discernment of digestion, absorption, metabolism of foods, and their influence on health.

A sample of free-living adult participants (N=25) was sought to validate the technology over 2 data collection periods of 14 days each (28 days total). A reference method was designed to measure dietary intake; all meals were prepared, calibrated, and served at a campus dining facility and consumed under the direct observation of a trained research team. Approval for the research study and protocol was obtained from the University of California, Davis (UC Davis), institutional review board.

### Participants

Participants aged 18 to 50 years were recruited from the UC Davis campus using emails and flyers. Those interested were screened by phone for inclusionary and exclusionary criteria. The exclusion criteria included historical or current diagnosis of chronic disease (including diabetes or prediabetes, cancer, asthma, hypertension, cardiovascular disease, stroke, kidney, thyroid, or autoimmune disease), known food allergies, current dieting or restricted dietary habits (ie, vegetarian, ketogenic, reduced calorie), pregnancy or lactation, smoking, drug or alcohol addiction, excessive exercise or athletic training, and taking medications impacting digestion or metabolism. In-person screenings were conducted at the Ragle Human Nutrition Center on the UC Davis campus. Participants who qualified after the phone screening were invited for in-person screening to complete a fasting blood draw, blood pressure, and anthropometric measurements. Copies of approved, signed consent forms were obtained from all participants at screening. All female participants completed urine pregnancy tests. Blood pressure measurements were obtained using a Nellcor pulse oximeter with OxiMax technology from Welch Allyn. For anthropometry, a digital scale by Scale-Tronix was used to weigh participants to the nearest 0.1 kg, and a wall stadiometer was used to measure height to the nearest 0.1 cm. Anthropometric measurements were used to calculate baseline BMI (weight [kg]/[height (m)]^2^). As the wristband was intended to measure nutrient intake in a weight-stable population over the study duration, individuals with fluctuating weight (>5 lbs over the previous month) were excluded. All anthropometric measurements were conducted by the principal investigator (PI) using methods defined in the anthropometric standardization reference manual [28]. Participants were assigned a study ID on enrollment, and all data collected were maintained private and deidentified. Monetary compensation was offered to each participant who completed the screening (US $10), phase 1 (US $125), and phase 2 (US $150).

For metabolic screening, blood was drawn into ethylenediaminetetraacetic acid and plasma separation lithium heparin blood collection tubes and immediately placed on ice. Within 2 hours of collection, blood samples were centrifuged at 1800×g for 15 min at 4°C to separate blood from plasma and frozen at −20°C until laboratory analyses were performed in batches. Blood samples were analyzed, and individuals who tested abnormally for metabolic health indicators including complete blood count, fasting blood glucose, hemoglobin A_1c_, erythrocyte sedimentation rate, serum protein, creatine, alkaline phosphatase, potassium, and carbon dioxide were excluded. Tests were performed according to the manufacturer’s instructions and quality controls by UC Davis Health System Medical Diagnostics.

Between August 2018 and September 2018, 76 adults were screened, and 35 met the inclusion criteria for enrollment in phase 1 of the study that would take place from September 25 to October 9, 2018. The initial sample included 20 women and 15 men, with an ethnic distribution of 38% white, 41% Asian, and 21% Hispanic, an average age of 25.3 (SD 6.4) years, and a mean BMI of 24.2 (SD 5.1) kg/m^2^. Three participants dropped out during the first week of phase 1 because of time constraints that prohibited multiple visits to the campus dining facility each day. Phase 1 was completed by 32 participants, of which 24 enrolled in phase 2 (October 30 to November 13, 2018). During phase 2, 2 participants completed 10 of 14 days because of scheduling conflicts and were included in the analyses.

### Data Collection

**Figure 1:**
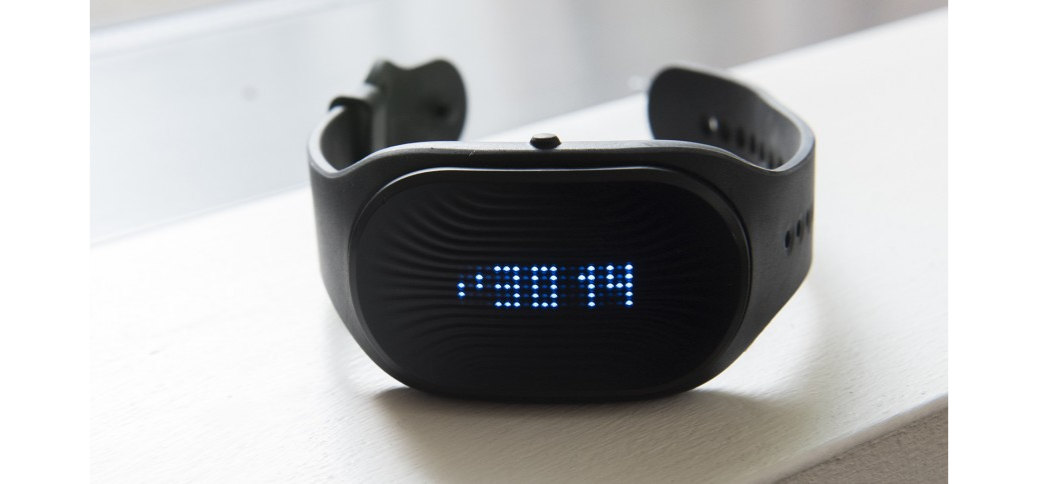
Healbe GoBe2 smartband.

Participants were assigned a GoBe2 (Figure 1) and instructed to use the latest version of the accompanying app synchronized to the wrist unit. The technology translates sensor signals into energy intake and expenditure outputs over a 24-hour period, in accordance with the rate of nutrient absorption. Participants received an explanation on how the wristband estimates personal calorie intake and expenditure throughout the day and over the week as well as its other functions, including heart rate, sleep, hydration, and stress measurement. Participants were instructed to synchronize the wrist unit with the app twice daily, in the morning and at night, and to collect screenshots from within the app that captured the previous day’s final energy (kcal) estimations. The screenshots were collected by research staff as records of daily caloric outputs, including daily intake, expenditure, and total balance.

### Quantification of Dietary Intake

A reference method was developed to quantify the daily food, calorie, and macronutrient intake of participants during the 2 study periods. The project team collaborated with UC Davis Dining Commons (DC), a series of dining facilities where campus residents primarily eat but are also open to the campus community and public. A strategy was developed to carry out the nutrition study within the university dining facility. In this approach, a specific *project menu* was created in coordination with the facility’s existing cycle menu serving all dining patrons. In this way, the dining facility’s normal operations were minimally perturbed, and the study team used the facility’s existing food prepared in accordance with standardized recipes from which nutritional information was readily derived. Meal cards were purchased for study participants and swiped on their arrival at each meal to deduct the meal price from the card. Student research assistants were trained to carry out food measurement at each meal, nutrient analysis, and data entry.

### Menu Planning

A registered dietitian (RD) on the research team collaborated with the dining facility’s primary chef to design the project menu. Menu items were selected to serve to study participants at breakfast, lunch, and dinner, using the following criteria: balanced macronutrients at each meal per USDA MyPlate guidelines and minimal multi-ingredient *mixed* dishes (ie, no casseroles, lasagna, pizza, etc). Mixed dishes were avoided to reduce error in calculating calories and macronutrients that were served at each meal. When necessary, menu modifications were requested to fit the study menu criteria (ie, sauces served on the side and sandwich ingredients served separately). Separating ingredients allowed the staff to weigh foods more precisely and calculate energy and macronutrient profiles accordingly.

### Energy and Macronutrient Analysis for Onsite and Offsite Food Consumption

Overall, 2 research staff were trained and designated to analyze each project menu item for energy (kcal) and macronutrient content. Items were analyzed using a combination of the USDA Food Composition Database and the dining facility’s nutritional database. In the latter, menu items were previously analyzed and recorded by the DC’s RD using either product nutritional labels (when available) or the USDA Food Composition Database. Each menu item was analyzed for serving size, calories, grams of protein, fat, and carbohydrate content per serving and scaled to 100 g.

The RD determined a *standard* serving size for each menu item (eg, 1 cup cooked oats, 1 cup vegetables, half cup beans, 4 oz lean protein, or three-fourth cup grain). Participants were not restricted to the standard serving sizes and were free to request more or less food portions to meet their individual dietary needs. All deviations from standard portions were recorded by the research staff for each participant.

The primary chef coordinated study meals according to the study menu preference. Each meal was prepared in a commercial kitchen on the UC Davis campus by trained food service personnel following a stringent hazard analysis critical control points (HACCP) protocol. All food was delivered to the designated research study area of the facility and received by a team of research staff for onsite portioning and serving to study participants. The study leads inspected each delivered menu item for accuracy, noting any deviations as needed.

Study participants arrived at the dining facility during scheduled breakfast, lunch, and dinner mealtimes. On arrival, they were greeted by research staff, and the meal was paid for at the door using preloaded meal cards. Each morning at breakfast, research staff collected daily information from participants, including paper records of offsite foods consumed in the previous 24 hours and details of wristband use (charging, removals, and reported problems). A brief daily in-person interview was conducted each morning to collect details on exercise, any perceived stress, water intake, defecation, and continuous glucose monitoring (CGM) skin contact in the previous 24 hours. At each meal, participants could request either the standard meal offering or certain menu items in more or fewer portions according to preference. The participants’ meal choices were recorded on paper meal slips that were delivered to research staff responsible for food portioning, plating, and weighing.

All project staff were trained by the RD in appropriate food handling and safety, food weighing, and meal recording duties. Before each meal, a team of research staff was briefed on how to portion and serve each menu item. Individual menu items were weighed and recorded (0.0 g) using calibrated food scales, portioned using standardized tools, and served at each onsite meal. Each dish with multiple food components was deconstructed into individual items and was weighed and recorded individually (ie, burgers were deconstructed to individually weigh patty, bun, cheese, ketchup, mustard, and tomato). Staff assumed various roles to ensure optimal meal-time efficiency (ie, *menu collector*, *food weigher*, and *data recorder*). After recording the weight of each food item and time of meal (00:00), the plate was served to the appropriate participant. Participants were encouraged to consume all food served at each meal, but this was not mandatory. The plate waste from each participant was deconstructed by ingredient and individually weighed at the end of each meal period.

After each participant finished eating, the research staff weighed and recorded each individual item left on the plate. The gram weight of each food item consumed was quantified and entered into an electronic database. Energy and macronutrient profiles of each menu item were obtained from the dining facility’s recipe, the food label, or the USDA Food Composition Database and calculated according to the gram weight consumed.

Consuming foods outside of the study facility was discouraged but not prohibited to minimize the changes made to the participants’ usual habits and metabolism. If food was consumed outside of the dining facility, participants were instructed to follow a specified procedure of self-reporting, including only consuming packaged foods, weighing and recording the weight of each individual food item, and providing the food label from the package. To minimize the miscalculation of nutrient intake of offsite foods, participants were provided with various packaged foods of known nutritional content (protein bars, jerky sticks, ramen noodles, fruit leather, and chocolate bars). They were asked to consume these foods; if this was not possible, they were required to photograph the food and record the food item, brand, time of consumption, and food weight (g) using a calibrated food scale and recording in a food diary. Offsite food diaries were collected daily from participants.

Participants unable to report to the dining facility for a scheduled meal time received alternative options and selected a prepackaged meal from a convenience market managed by the dining facility. Nutritional information from the item was extracted from the nutrition label and recorded. A team of staff recorded and analyzed the nutritional values of all offsite foods consumed by the participants. Information from the food intake data of 1 participant, as measured by the study reference method, is presented in Table 1.

**Table 1:** Daily food intake record of 1 study participant.

Time of meal (00:00)	Menu item	Amount consumed (g)	Energy intake (kcal)	Source of nutrition information
9:34 AM	Scrambled eggs	53	69	Product label^a^
9:34 AM	Cooked oatmeal	189	105	Product label^a^
9:34 AM	Blueberries	69	39	USDA^b^ Food Composition Database
9:34 AM	Bacon	15	56	USDA Food Composition Database
9:34 AM	Milk 1%	0	0	Product label
9:34 AM	Coffee, fresh brewed	246	0	N/A^c^
9:34 AM	Granulated sugar	10	23	USDA Food Composition Database
2:11 PM	Bun	90	218	Product label^a^
2:11 PM	Beef, ground, cooked	125	156	Product label^a^
2:11 PM	Sauce	40	53	Product label^a^
2:11PM	Mixed greens	57	16	USDA Food Composition Database
2:11 PM	Artichoke hearts, canned	21	6	USDA Food Composition Database
2:11 PM	Cherry tomatoes	44	12	USDA Food Composition Database
2:11 PM	Cucumbers, sliced	51	5	USDA Food Composition Database
2:11 PM	Carrots, shredded	25	10	Product label
2:11 PM	Olive oil	12	96	Product label
2:11 PM	Balsamic vinegar	19	17	Product label
6:46 PM	Chicken tamales	304	669	Product label^a^
6:46 PM	Vegetables, roasted	250	143	Product label^a^
6:46 PM	Rice, cooked	61	105	Product label^a^
6:46 PM	Milk 1%	0	0	Product label
11 AM	Energy bar	52	210	Product label
1 PM	Energy bar	48	190	Product label
8 PM	Dehydrated soup	64	290	Product label
10 PM	Energy bar	68	290	Product label
N/A	N/A	1913^d^	2753^d^	N/A

^a^Nutritional information of food items prepared by the University of California, Davis, Dining Commons.

^b^USDA: United States Department of Agriculture.

^c^N/A: not applicable.

^d^Final row contains column totals where applicable.

### Quality Assurance

Before this study, the PI conducted a series of small pilot trials over 1 year to inform this study design and data collection procedures using the wristband technology. During these pilot trials, it was observed that the form factor of the technology was the main barrier to collecting consistent, uninterrupted data during the postprandial digestion period that lasts several hours beyond each meal. Practically, any signal interruption during the meal or in the hours following it would result in loss of data and underestimation of calorie intake by the technology. Unfortunately, signal interruption occurred often and for a variety of reasons in this study; for example, periodic loss of contact with the skin was likely depending on the user’s wrist size and shape. In addition, the wristband required an hour each day to obtain a full charge; any loss of charge would disable data collection accordingly. Several strategies were used to mitigate these challenges with the form factor. Participants were instructed to charge the wristband fully before any meal to avoid missing food intake and its subsequent digestion (ie, charging band in the morning before consuming food for the day). It was acceptable to charge the wristband at any point during the day as long as no food had been consumed for 3 hours prior. On arriving at the first meal of the day, the research staff visually confirmed that the wristband was positioned on each participant, such that the sensor was in complete and constant contact. Research staff used a third-party site (Dietitian’s Cabinet) to access participants’ deidentified data up to the minute from which the frequency of contact interruptions could be assessed. Those who had significant interruptions were targeted for individual solutions to improve sensor contact with the wrist, for example, tightening the wristband to achieve optimal sensor positioning.

Continuous blood glucose was monitored as a strategy to measure and account for nonadherence to the study’s dietary intake reporting protocols. The FreeStyle Libre (FSL) Pro System (Abbott Diabetes Care Inc) CGM system includes a unit with a water-resistant sensor that attaches to the back of the user’s upper arm. Within the unit is an Enlite sensor that consists of a wire containing glucose oxidase at the tip that is inserted subcutaneously with a dedicated inserting device. Glucose oxidase catalyzes a biochemical reaction in the presence of glucose and oxygen, which transfers electrons to a receiving molecule and creates a current that can be measured and converted into a glucose concentration [27]. The FSL Pro System collects up to 14 days of glucose readings, with recordings every 15 min. A single reader can be used to activate glucose data recording and download reports from multiple devices simultaneously. One study showed that the FSL’s mean absolute relative difference compared with measured capillary blood glucose levels was 13.2% (95% CI 12.0% to 14.4%) [29].

CGM sensors were secured to the tricep or rotator cuff region of participants’ arms on day 1 of the study, in the morning before consuming food or beverages. During the 14-day test period, units would occasionally become detached. The research staff downloaded data files from the participants’ sensors every 2 days to minimize any data loss. Text file reports were exported through the LibreView software program (Abbott Diabetes Care Inc, 2018) and a secure cloud-based system. CGM data were analyzed to assess the adherence of individual participants to reference dietary intake reporting protocols. Significant glucose increases (>20 mg/dL per 30 min) occurring outside of specified study mealtimes or not reported in food intake diaries were flagged for further examination.

### Statistical Analysis

The Bland-Altman analysis was conducted to compare daily energy intake (kcal/day) estimated by both the reference method and the wristband technology. Regression analyses were used to examine trends in the data and sample characteristics. Statistics were conducted in Microsoft 2008 (version 12.3.1) and Prism 8 2019 (version 8.3.1).

## Results

This study developed a dietary intake reference method to evaluate a wearable sensor with the potential to generate objective and precise data on the dietary intake of adult individuals. The data accuracy and practical application of the current GoBe2 model was interrogated over two 14-day test periods in an intended sample of 25 participants. Of the 35 participants who were originally enrolled in phase 1 of the study, 304 measurements (kcal/day) collected from 24 participants were retained from phase 2 after data cleaning to remove missing or aberrant values.

Of the total cases, 10.9% (33/304) were excluded because they lacked an accompanying set of complete CGM data for the 24-hour period. Of the remaining cases, 22.1% (60/271) had at least one event per day of rapid blood glucose increase that was inconsistent with the recorded meal time. Of those, 68.3% (41/60) were attributed to reported bouts of exercise or other physical activity. Although CGM was used to measure nonadherence to dietary intake reporting protocols, these data were not incorporated in the present dataset.

**Figure 2:**
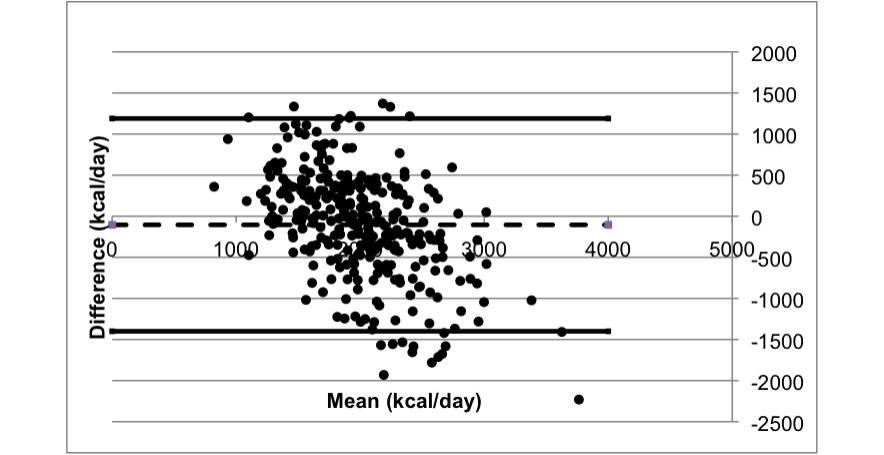
Bland-Altman (mean difference) plot of estimated nutrient intakes (kilo/day) by the test and reference method (N=304). Solid lines represent upper-lower limits of agreement, and the dashed line represents bias.

As depicted in Figure 2, a Bland-Altman analysis showed a mean bias of −105 (SD 660) kcal/day, with 95% limits of agreement (LoA) between −1400 and 1189. Pearson correlation coefficient between the 2 methods was r=−0.496 (95% CI −0.576 to −0.406; *P*<.001). Linear regression analysis on the Bland-Altman plot revealed a regression equation of Y=−0.3401X+1963 that was significant (*P*<.001). A multiple regression analysis was conducted with the participants’ age, sex, and BMI classification as independent confounding variables, but no significant effects were seen on the bias. Analysis of variance tests were conducted to assess the effects of the participants’ age, sex, and BMI classification on bias, and the effects were not significant (*P*=.15, .18, and .12, respectively).

## Discussion

Negative bias in the Bland-Altman analysis indicated a general underestimation of daily calorie intake by the wristband compared with the reference method. Despite a relatively small bias, the LoAs were wide, making the results of the comparison ambiguous. Regression analyses indicated a tendency for the wristband to systematically overestimate for lower calorie intake and underestimate for higher intake.

Our preliminary validation results indicate that although the ability of GoBe2 to make phenotypic discernments responsive to diet by noninvasive means has wide-reaching utility in research and practice, notable feasibility challenges were observed for free-living study participants to reliably use the technology to achieve accurate and precise measurements. These challenges were largely attributed to limitations in the technology’s form factor. In observation, when positioned correctly on the arm and fully charged, the wristband’s calorie intake estimates generally appeared accurate and provided interesting visuals pertaining to the body’s rate of nutrient absorption. However, to achieve precise detection and accurate estimation of dietary intake, the unit’s sensor required adequate skin contact be maintained at all times. Achieving this proved to be a considerable challenge for several reasons, including (1) battery life, as the unit required an hour of charging each day, which required removal of the device, preventing the detection of calories ingested within several hours before removal; (2) the wristband’s bulky size, dimensions, and/or appearance were challenges for some users to maintain comfort and position on the arm; and (3) the user’s own wrist size and shape; for example, small or tapered wrists were likely to result in inconsistent sensor contact. As described previously, several strategies were included in the study design to prevent data loss, such as targeting problematic cases early, checking in with participants, and monitoring sensor position daily. However, data loss from poor sensor contact was a significant barrier to the technology’s ability to reliably detect calorie intake. Separate analyses, not reported in this study, further examine the technology’s efficacy using data collected only during periods of protocol adherence concerning food reporting and technology use.

Establishing reliable adherence or compliance protocols is a widespread goal in measuring the dietary intake of human subjects [23]. Continuous glucose monitors were used to measure the participants’ adherence to food intake recording protocols. Although CGM data do not provide a direct measure of dietary intake, its measurement of the body’s relative physiological response to food intake can serve as a proxy to identify inconsistencies in reported intake data and blood glucose activity. Examination of CGM data confirmed that although a few participants (n=2) were likely nonadherent with dietary intake reporting protocols, aberrant increases in blood glucose levels could be attributed to multiple factors including exercise or other bouts of physical activity. The authors concluded that complex outcomes on CGM measurement and the participants’ adherence would be appropriately detailed in the context of measuring or impacting compliance in nutrition research. Some challenges to using the CGM devices to collect data over continuous 14 days were also related to form factor limitations. The sensor included an adhesive material attached to the skin, but some devices became dislodged during the 14-day study period (13/72, 18% CGMs attached), causing complete or partial data loss. Of the 24 participants, 2 (8%) had repeated CGM sensor displacement, which was more likely to occur during physical activity (biking, gym workout, and weight lifting) and/or excessive sweating. In these cases, a skin adhesive (Skin-Tac) was useful in reinforcing the CGM attachment. As the FSL Pro System did not include individual readers with each unit, participants were blinded to their personal glucose data. At the end of the study period, data reports summarizing glucose patterns were generated and distributed to participants. Readouts included daily blood glucose averages (g/ dL) across each 24-hour period, average glucose trend lines across each 24-hour period, and likelihoods of hypoglycemia or hyperglycemia during specified windows. A total of 7 days’ worth of daily blood glucose trend lines were color coded and superimposed onto summary graphs. Participants were provided with general guidance from the RD to interpret numerical data into a relevant and actionable context for health and diet.

By collaborating with the university facility, this study used existing food production operations, resources, and personnel to carry out an extensive dietary observation study. Despite numerous strengths in the study design and utilization of a novel research environment, limitations were revealed during project implementation. For example, in the food facility where dishes were prepared for high throughput mass consumption, the exact quantity of nutrients in each portion could not be consistently and routinely ensured using these methods alone. In addition, considering that the project targeted students on a university campus, protocol adherence was less than anticipated, particularly with regard to meal attendance. Of the 42 total study meals offered to each of the 24 participants during the second 14-day testing period (1008 total meals), 56% of the scheduled meals were attended (565 meals). To improve adherence in the future, stricter enforcement of meal attendance is recommended. Studies excluding offsite food consumption may help improve the accuracy of nutrient intake reporting, with strategies in place to account for protocol adherence. Given that numerous factors were involved with intermittent data loss from the wristband technology, 2 weeks was defined as the minimum period required to gather continuous data from 25 free-living participants for validation purposes. Longer study periods could affect adherence issues without stricter guidelines around participant meal attendance.

This study validated participants’ calorie intake as recorded by the wearable device, in comparison with a reference diet. The deviations in and between methods could be explained by any combination of the following factors: form factor limitations (skin contact/battery); the participants’ nonadherence to dietary protocol (ie, consuming and failing to report ingested food or drink); interindividual differences in measured intake versus actual nutrient absorption and metabolism; human error in calculating food intake using the USDA Database; potential deviations from the standardized recipe during the meal preparation process; inability of the USDA Database account for nutrient variation depending on food ripeness, geographic location, and soil quality; inherent data loss because of required 1-hour daily device charging periods; and inaccuracies pertaining to technology algorithm development. Future studies should incorporate these suggestions for improvement to further interrogate the potential of wearable devices to accurately capture caloric and macronutrient intake. Ongoing engineering adjustments are recommended to accurately estimate the energy and nutrient intakes of individuals consuming various diets.

Tools are urgently needed to obtain accurate and precise measurements of diet and nutrition. Enhancing knowledge about individual phenotypes allows for more precise and predictive dietary guidance and intervention, and this has the potential to transform how people make informed diet and lifestyle choices. In today’s personalized marketplace, we routinely use sophisticated technology to acquire personalized step-by-step guidance that assures arrival at nearly any physical destination (eg, satellite navigation). In accordance with the natural diversity of humans as unique phenotypes, this concept could also be applicable to the realm of food and diet. In other words, there is a need for sensitive and specific devices to deliver step-by-step directions to any desired *health destination*. This requires the tools able to quantify health status and progress over important time scales and adjust trajectories according to biofeedback. Smartphones are the cornerstone of the customization and precision of modern life, incorporating precise personal information with global databases accessible through cloud storage and applying straightforward computational algorithms to guide decisions. This basic principle and its applications offer a sophisticated and diverse range of possibilities for enhancing our individual experience, whether through personalized navigation, physical activity tracking, tailoring fitness routines, and identifying a song or even a face. However, to date, the app market does not offer reliable solutions for automating the quantification of dietary intake that would significantly impact individualized quality of life decisions. Measurement and tracking devices provide practical utility for discerning phenotypic traits and defining progressive *roadmaps to personalized health destinations*. Automated nutrient tracking devices could precisely inform diet and lifestyle choices appropriate to health status and guide individuals toward desired goals, including everything from diet planning to cardiometabolic performance. Validation and effectiveness testing of candidate devices are essential steps to be taken for the use of precision technologies to inform personalized diet and lifestyle guidance.

### Conclusions

This study documented high variability in both the utility and accuracy of a wristband sensor to track nutritional intake (kcal/day). The researchers acknowledge that because dietary intake measurement of individuals has inherent challenges related to accuracy and variability, achieving precision of reference methods is a notable challenge. This study highlights the need for innovative measurement tools that are precise, reliable, and efficacious to facilitate accurate personalized dietary measurement.

## Abbreviations

CGM: continuous glucose monitoring
DC: dining commons
FSL: FreeStyle Libre
LoA: limits of agreement
PI: principal investigator
RD: registered dietician
UC Davis: University of California, Davis
USDA: United States Department of Agriculture
